# Modeling the decarburization of expansion droplets based on the solid phase ratio of slag and data fitting during BOF steelmaking process

**DOI:** 10.1038/s41598-025-01424-1

**Published:** 2025-05-10

**Authors:** Zi-cheng Xin, Wen-hui Lin, Jiang-shan Zhang, Kai-xiang Peng, Qing Liu

**Affiliations:** 1 State Key Laboratory of Advanced Metallurgy, University of Science and Technology Beijing, Beijing, 100083 China; 2 School of Automation and Electrical Engineering, University of Science and Technology Beijing, Beijing, 100083 China; 3 Jiangsu Jinheng Information Technology Co., Ltd, Nanjing, 210031 Jiangsu China

**Keywords:** BOF steelmaking, Metal droplets, Solid phase ratio, Decarburization comprehensive model, Engineering, Chemical engineering, Applied mathematics

## Abstract

In the process of basic oxygen furnace steelmaking, the high-velocity jet impacts the molten metal pool, resulting in the generation of splashing metal droplets with varying particle sizes. The expansion decarburization of metal droplets in the emulsion phase is one of the main steel-slag reaction behaviors. In this paper, firstly, a theoretical decarburization model of expansion droplet was established, finding biases in their calculation results. Then, the solid phase ratio in different FeO content of slag was analyzed, demonstrating its significant impact on the reactivity performance of slag. Finally, an improved decarburization comprehensive model for expanded droplets, based on the solid phase ratio of slag and data fitting, was developed and validated using experimental data. The results indicate that the improved droplet decarburisation comprehensive model shows a good agreement with the experimental data in predicting changes in carbon content, with Pearson correlation coefficient exceeding 0.94 for different FeO contents. ‌For FeO contents of 3%, 10%, 20%, and 30%, the model achieves MAE (0.035%, 0.024%, 0.056%, 0.059%) and RMSE (0.0034%, 0.0019%, 0.011%, 0.018%), respectively. This model can accurately calculate the change of carbon content of metal droplets under various complex multiphase slag conditions.

## Introduction

Basic oxygen furnace (BOF) steelmaking is currently the most important steel production method in the world. The global production of BOF steel accounted for more than 70% of the total steelmaking production in 2022^1^. In the context of promoting intelligent manufacturing in the steel industry, the realization of modeling and intelligent control of BOF steelmaking has become imperative^2^. During the BOF smelting process, metal droplets and bubbles move in the slag, resulting in emulsification of the slag. Meanwhile, the metal droplets increase the contact surface area for slag-steel reactions, thereby promoting the progress of slag-metal reactions. In addition, the expansion decarburization of metal droplets in the emulsion phase is one of the main slag-metal reaction behaviors. Therefore, it is necessary to conduct research on the decarburization model of expanded droplets.

Decarburization and heating are the two most critical metallurgical tasks of BOF steelmaking, involving the physical interactions and chemical reactions among the gas, solid, and liquid phases within the furnace. Metallurgists have conducted extensive studies on the BOF steelmaking process. Shao et al.^3^ established a hybrid model for BOF oxygen blowing time prediction based on oxygen balance mechanism and deep neural network, achieving good prediction results. Dong et al.^4^ developed a dynamic control model of basic oxygen furnace last blowing stage based on improved conditional generative adversarial network, and the actual data from two steel grades were used to evaluate the effectiveness of this model. Mitas et al.^5^ researched the distribution of oxygen between the gaseous and liquid oxidation products at the hot spot in BOF steelmaking. Singha^6^ established a dynamic control model using FactSage and its macro program to analyze the contribution of emulsion zone in refining of basic oxygen steelmaking converter. Rout et al.^7^ developed a dynamic decarburization model of basic oxygen steelmaking process based on multizone reaction kinetics, and the model predictions demonstrate good agreement with industrially measured data. During decarburization and heating process, slag emulsification continuously occurs, with metal droplets and gas bubbles dispersed within the slag, forming an intensely mixed emulsified phase. The metal droplets in the emulsified phase have a much larger specific surface area than the molten metal in the bath, significantly increasing the contact area for the slag-metal reactions, which is one of the key reasons for the high reaction rate in the BOF. Experimental studies^8,9^ showed that increasing the top-blowing flow rate intensifies surface instability and droplet splashing. The Weber number and Kelvin–Helmholtz instability theory were used to analyse the mechanism of droplet formation due to gas–liquid impact^10–12^.

With the advancement of experimental techniques, there has been a deeper understanding of the dynamic behavior of metal droplets in slag. Mulholland et al.^13^ first observed the gas halo phenomenon during the reaction of Fe–C droplets with slag using X-ray imaging technique. The results indicated that the faster the decarburization rate, the larger the gas halo and the greater the apparent diameter of the droplets. Subsequently, Gaye and Riboud^14^ analyzed the morphological changes of droplets and concluded that the large amount of CO generated inside the droplets caused emulsification and expansion of the droplets during the rapid decarburization stage. Sun et al.^15^ investigated the droplet splashing behavior in steelmaking converter based on VOF-to-DPM hybrid model and AMR technique, and clarified the generation mechanisms of splashing droplets. Molloseau and Fruehan^16^ found that the emulsified droplets remained suspended in foamy slag for about 8 s before settling back into the non-emulsified slag. During this suspension, the decarburization rate of the metal droplets was extremely high. As the decarburization rate decreased to a certain level, the emulsification of the droplets gradually disappeared, and they settled back into the slag. Coley et al.^17,18^ proposed a nucleation rate formula for the CO bubbles generated inside the droplets, and the mass transfer coefficient of droplet containing carbon was significantly higher than that of droplets without decarbonization. Brooks et al.^19^ obtained the general relationship between droplet carbon content and time through nonlinear fitting. However, the empirical model of data fitting is only applicable to a few specific conditions. Additionally, studies^20,21^ explored the heat transfer mechanisms of expansion droplets in oxygen steelmaking. The accurate calculation of carbon content of metal droplets under various complex multiphase slag conditions remains a subject to be further studied^22^.

This research aims to develop a droplet decarburization model that can be used to accurately estimate the variation of carbon content in BOF steelmaking process. Firstly, the theoretical decarburization model of expansion droplet was established. Then, the solid phase ratio of slag was analyzed in different FeO content of slag. Finally, an improved decarburization comprehensive model for expansion droplet was developed and validated using experimental data. This study can accurately calculate the change of carbon content of metal droplets under multiphase slag conditions, and is of great significance to realize the modelling and intelligence in BOF steelmaking processes.

## Analysis of theoretical decarburization model of expansion droplet

In BOF steelmaking, the supersonic oxygen jet impinges on the liquid surface of the molten metal bath from top to bottom, forming an impact crater and the gas flow rebounds upward. The metal liquid at the edge of the crater surface of the molten bath is torn by the shear force of the rebound gas flow, resulting in the metal droplets splashing into the emulsion phase above the molten bath. In this section, the theoretical decarburization model was established and verified using experimental data from the literature.

For Fe–C droplets without solidification, they always remain liquid or emulsified state in the slag. Therefore, the decarburization reaction of the droplets can continue sustainably until they descend back into the molten metal bath. The final carbon content of the droplets will be closer to the equilibrium carbon content ($w_{{\text{[C]}}}^{*}$) of the slag-metal reaction. However, the restrictive link of decarburization reaction is different for the slag state with different FeO content and the droplets with different carbon content. Therefore, in order to calculate the decarburization of droplets, it is necessary to judge the mass transfer rate of carbon ($J_{{\text{C}}}$) in liquid metal and the mass transfer rate of FeO ($J_{{{\text{FeO}}}}$) in slag.

Given the known kinematic velocity of the droplets, the effective mass transfer coefficient for the transfer of carbon from the metal droplets to the reaction interface can be calculated using Higbie’s penetration theory^19^, as shown in Eq. (1). The mass transfer rate of carbon in the metal droplet can be determined by Eq. (2).

1 $$k_{{\text{C}}}^{{{\text{eff}}}} { = }2\sqrt {\frac{{D_{{\text{C}}} u_{{\text{d}}} }}{\pi d}}$$

where, $k_{{\text{C}}}^{{{\text{eff}}}}$ is the effective mass transfer coefficient of carbon in the metal droplet, m/s; $D_{{\text{C}}}$ is the diffusion coefficient of carbon in the metal droplet, m^2^/s; $u_{d}$ is the kinematic velocity of the droplet, m/s; *d* is the apparent diameter of the droplet, m.

2 $$J_{{\text{C}}} = k_{{\text{C}}}^{{{\text{eff}}}} \cdot A_{{\text{d}}} \cdot \left( {C_{{\text{[C]}}}^{{\text{b}}} - C_{{\text{[C]}}}^{*} } \right) = k_{{\text{C}}}^{{{\text{eff}}}} \cdot A_{{\text{d}}} \cdot \rho_{{\text{d}}} \cdot \frac{{\left( {w_{{\left[ {\text{C}} \right]}}^{{\text{b}}} - w_{{\left[ {\text{C}} \right]}}^{*} } \right)}}{{100 \cdot M_{{\text{C}}} }}$$

where, $J_{{\text{C}}}$ is the mass transfer rate of carbon in the metal droplet, mol/s; $A_{{\text{d}}}$ is the surface area of the metal droplet, m^2^; $C_{{\text{[C]}}}^{{\text{b}}}$ is the molar concentration of carbon for liquid metal, mol/m^3^; $C_{{\text{[C]}}}^{*}$ is the molar concentration of carbon at the reaction interface, mol/m^3^; $w_{{\left[ {\text{C}} \right]}}^{{\text{b}}}$ is the mass concentration of carbon for liquid metal, %; $w_{{\left[ {\text{C}} \right]}}^{*}$ is the mass concentration of carbon at the reaction interface, %; $M_{{\text{C}}}$ is the molar mass of carbon, kg/mol; $\rho_{{\text{d}}}$ is the density of metal droplet, kg/m^3^.

Similarly, the effective mass transfer coefficient and the mass transfer rate of FeO in the slag can be derived, as shown in Eqs. (3) and (4).

3 $$k_{{{\text{FeO}}}}^{{{\text{eff}}}} { = }2\sqrt {\frac{{D_{{{\text{FeO}}}} u_{d} }}{\pi d}}$$

4 $$J_{{{\text{FeO}}}} = k_{{{\text{FeO}}}}^{{{\text{eff}}}} \cdot A_{{\text{d}}} \cdot \rho_{{\text{s}}} \cdot \frac{{\left[ {w_{{\left( {{\text{FeO}}} \right)}}^{{\text{b}}} - w_{{\left( {{\text{FeO}}} \right)}}^{*} } \right]}}{{100 \cdot M_{{{\text{FeO}}}} }}$$

where, $J_{{{\text{FeO}}}}$ is the mass transfer rate of FeO in the slag, mol/s; $k_{{{\text{FeO}}}}^{{{\text{eff}}}}$ is the effective mass transfer coefficient of FeO in the slag, m/s; $D_{{{\text{FeO}}}}$ is the diffusion coefficient of FeO in the slag, m^2^/s; $w_{{\left( {{\text{FeO}}} \right)}}^{{\text{b}}}$ is the mass concentration of FeO for slag, %; $w_{{\left( {{\text{FeO}}} \right)}}^{*}$ is the mass concentration of FeO at the reaction interface, %; $M_{{{\text{FeO}}}}$ is the molar mass of FeO, kg/mol; $\rho_{{\text{s}}}$ is the density of slag, kg/m^3^.

The ratio of the mass transfer rates of FeO and C is shown in Eq. (5).

5 $$\frac{{J_{{{\text{FeO}}}} }}{{J_{{\text{C}}} }}{ = }\frac{{k_{{{\text{FeO}}}}^{{{\text{eff}}}} }}{{k_{{\text{C}}}^{{{\text{eff}}}} }} \cdot \frac{{\rho_{{\text{s}}} }}{{\rho_{{\text{d}}} }} \cdot \frac{{M_{{\text{C}}} }}{{M_{{{\text{FeO}}}} }} \cdot \frac{{\left[ {w_{{\left( {{\text{FeO}}} \right)}}^{{\text{b}}} - w_{{\left( {{\text{FeO}}} \right)}}^{*} } \right]}}{{\left( {w_{{\left[ {\text{C}} \right]}}^{{\text{b}}} - w_{{\left[ {\text{C}} \right]}}^{*} } \right)}}$$

It is assumed that there are critical transition points $R_{{{\text{transit}}}}^{{{\text{const}}}}$ and $R_{{{\text{transit}}}}^{T}$: when $\left( {{{J_{{{\text{FeO}}}} } \mathord{\left/ {\vphantom {{J_{{{\text{FeO}}}} } {J_{{\text{C}}} }}} \right. \kern-0pt} {J_{{\text{C}}} }}} \right) \le R_{{{\text{transit}}}}^{{{\text{const}}}}$, all carbon is produced into CO ($P_{{{\text{CO}}}} { = }1$), and the decarburization rate is controlled by mass transfer of FeO in the slag; When $R_{{{\text{transit}}}}^{{{\text{const}}}} < \left( {{{J_{{{\text{FeO}}}} } \mathord{\left/ {\vphantom {{J_{{{\text{FeO}}}} } {J_{{\text{C}}} }}} \right. \kern-0pt} {J_{{\text{C}}} }}} \right) < R_{{{\text{transit}}}}^{T}$, the chemical reaction produces CO and CO_2_ at the same time. The partial pressure ratio (${{P_{{{\text{CO}}}} } \mathord{\left/ {\vphantom {{P_{{{\text{CO}}}} } {P_{{{\text{CO}}_{{2}} }} }}} \right. \kern-0pt} {P_{{{\text{CO}}_{{2}} }} }}$) is related to ${{J_{{{\text{FeO}}}} } \mathord{\left/ {\vphantom {{J_{{{\text{FeO}}}} } {J_{{\text{C}}} }}} \right. \kern-0pt} {J_{{\text{C}}} }}$, and the decarburization rate is controlled by mass transfer of carbon in the metal droplet. When $\left( {{{J_{{{\text{FeO}}}} } \mathord{\left/ {\vphantom {{J_{{{\text{FeO}}}} } {J_{{\text{C}}} }}} \right. \kern-0pt} {J_{{\text{C}}} }}} \right) > R_{{{\text{transit}}}}^{T}$, the chemical reaction simultaneously generates CO and CO_2_ in a certain proportion ($P_{{{\text{CO}}}} < 1$, the ${{P_{{{\text{CO}}}} } \mathord{\left/ {\vphantom {{P_{{{\text{CO}}}} } {P_{{{\text{CO}}_{{2}} }} }}} \right. \kern-0pt} {P_{{{\text{CO}}_{{2}} }} }}$ is constant, and the partial pressure ratio constant is different at different reaction temperatures), and the decarburization rate of the reaction is controlled by the mass transfer of carbon in metal droplets.

**Fig. 1:**
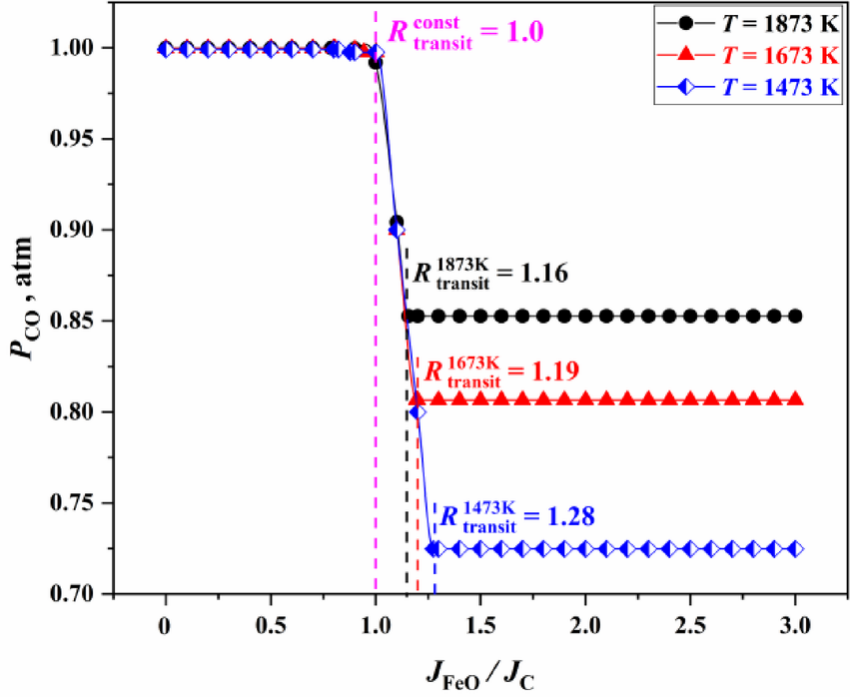
Changes of equilibrium partial pressure ($P_{{{\text{CO}}}}$) under different $\left( {{{J_{{{\text{FeO}}}} } \mathord{\left/ {\vphantom {{J_{{{\text{FeO}}}} } {J_{C} }}} \right. \kern-0pt} {J_{C} }}} \right)$.

In this study, the Equilib module of FactSage 7.0 thermodynamic software was used to calculate the changes of equilibrium partial pressure ($P_{{{\text{CO}}}}$) under different $\left( {{{J_{{{\text{FeO}}}} } \mathord{\left/ {\vphantom {{J_{{{\text{FeO}}}} } {J_{C} }}} \right. \kern-0pt} {J_{C} }}} \right)$, the changes of equilibrium partial pressure ($P_{{{\text{CO}}}}$) and partial pressure ratio (${{P_{{{\text{CO}}}} } \mathord{\left/ {\vphantom {{P_{{{\text{CO}}}} } {P_{{{\text{CO}}_{{2}} }} }}} \right. \kern-0pt} {P_{{{\text{CO}}_{{2}} }} }}$) under different temperatures, and the relationship between critical transition point $R_{{{\text{transit}}}}^{T}$ and temperature, as shown in Fig. 1.

In Fig. 1, $R_{{{\text{transit}}}}^{{{\text{const}}}}$ is the first transition point of the gas phase product partial pressure ratio, and it is also the transition point of the reaction rate restrictive link. $R_{{{\text{transit}}}}^{{{\text{const}}}}$ is identical to 1, and $R_{{{\text{transit}}}}^{T}$ is the second transition point of the gas phase product partial pressure ratio. When $\left( {{{J_{{{\text{FeO}}}} } \mathord{\left/ {\vphantom {{J_{{{\text{FeO}}}} } {J_{{\text{C}}} }}} \right. \kern-0pt} {J_{{\text{C}}} }}} \right) \le R_{{{\text{transit}}}}^{{{\text{const}}}}$, all carbon is produced into CO. When $\left( {{{J_{{{\text{FeO}}}} } \mathord{\left/ {\vphantom {{J_{{{\text{FeO}}}} } {J_{{\text{C}}} }}} \right. \kern-0pt} {J_{{\text{C}}} }}} \right) > R_{{{\text{transit}}}}^{T}$, the chemical reaction simultaneously generates CO and CO_2_ in a certain proportion. $R_{{{\text{transit}}}}^{T}$ is related to reaction temperature and decreases with the increase of temperature. When the temperature is 1873 K, $R_{{{\text{transit}}}}^{T}$ is 1.16.

According to the above analysis, the calculation formulas of the decarbonization rate under different control mechanisms can be obtained, as shown in Eq. (6).

6 $$\frac{{{\text{d}}w_{{\left[ {\text{C}} \right]}} }}{{{\text{d}}t}} = \left\{ \begin{gathered} \, J_{{{\text{FeO}}}} \cdot \frac{{M_{{\text{C}}} }}{{\rho_{{\text{d}}} \cdot V_{{\text{d}}} }} \, , \, \left( {{{J_{{{\text{FeO}}}} } \mathord{\left/ {\vphantom {{J_{{{\text{FeO}}}} } {J_{{\text{C}}} }}} \right. \kern-0pt} {J_{{\text{C}}} }}} \right) \le 1 \hfill \\ \, J_{{\text{C}}} \cdot \frac{{M_{{\text{C}}} }}{{\rho_{{\text{d}}} \cdot V_{{\text{d}}} }} \, , \, \left( {{{J_{{{\text{FeO}}}} } \mathord{\left/ {\vphantom {{J_{{{\text{FeO}}}} } {J_{{\text{C}}} }}} \right. \kern-0pt} {J_{{\text{C}}} }}} \right) > 1 \hfill \\ \end{gathered} \right.$$

Equation (2) and (4) are substituted into Eq. (6), and the decarbonization rate formula is obtained, as shown in Eq. (7).

7 $$\frac{{{\text{d}}w_{{\left[ {\text{C}} \right]}} }}{{{\text{d}}t}} = \left\{ \begin{gathered} \, \frac{1}{50} \cdot \sqrt {\frac{{D_{{{\text{FeO}}}} u_{{\text{d}}} }}{{\pi d^{3} }}} \cdot \frac{{\rho_{{\text{s}}} }}{{\rho_{{\text{d}}} }} \cdot \left[ {w_{{\left( {{\text{FeO}}} \right)}}^{{\text{b}}} - w_{{\left( {{\text{FeO}}} \right)}}^{*} } \right] \, , \, \left( {{{J_{{{\text{FeO}}}} } \mathord{\left/ {\vphantom {{J_{{{\text{FeO}}}} } {J_{{\text{C}}} }}} \right. \kern-0pt} {J_{{\text{C}}} }}} \right) \le 1 \hfill \\ \, \frac{3}{25} \cdot \sqrt {\frac{{D_{{\text{C}}} u_{{\text{d}}} }}{{\pi d^{3} }}} \cdot \left( {w_{{\left[ {\text{C}} \right]}}^{{\text{b}}} - w_{{\left[ {\text{C}} \right]}}^{*} } \right) \, , \, \left( {{{J_{{{\text{FeO}}}} } \mathord{\left/ {\vphantom {{J_{{{\text{FeO}}}} } {J_{{\text{C}}} }}} \right. \kern-0pt} {J_{{\text{C}}} }}} \right) > 1 \hfill \\ \end{gathered} \right.$$

**Fig. 2:**
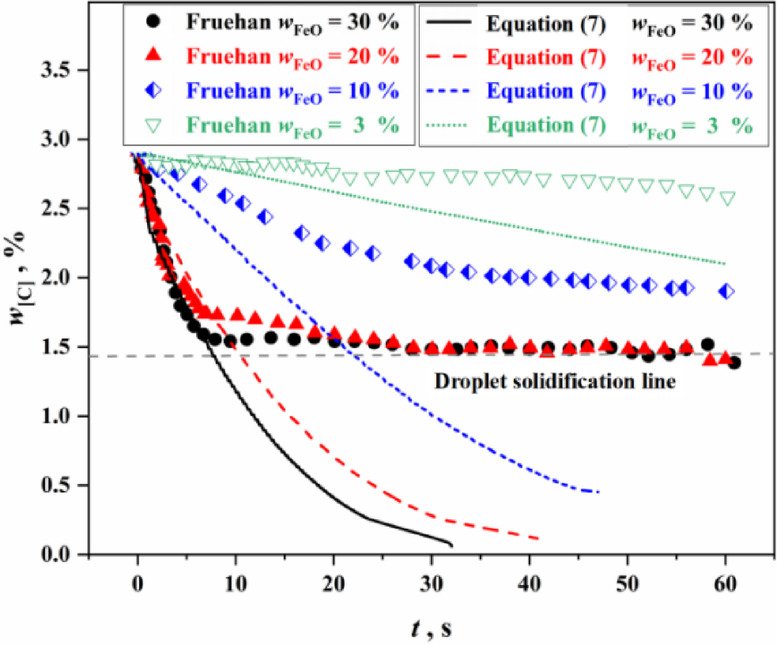
Comparison between the predicted value of the model proposed in this study and the experimental value.

The instant decarburization rate of metal droplets in emulsified slag under different conditions can be obtained from Eq. (7). According to Fruehan’s experimental conditions (Synthetic slag: 60g, ${{w_{{{\text{CaO}}}} } \mathord{\left/ {\vphantom {{w_{{{\text{CaO}}}} } {w_{{{\text{SiO}}_{{2}} }} }}} \right. \kern-0pt} {w_{{{\text{SiO}}_{{2}} }} }} = 1.2$, $w_{{{\text{MgO}}}} = 12 \, \%$; Fe–C alloy pellet: 1g, *w*_[C]_ = 2.19%), the relevant experimental parameters were substituted into Eq. (7), and the comparison results between the carbon content change curve predicted by the model and the experimental data values were obtained, as shown in Fig. 2.

In Fig. 2, two situations can be observed: (1) For slag conditions with 20% and 30% FeO content, the prediction results of theoretical decarbonization model are in good agreement with the results of Fruehan experiment (the part above the droplet solidification line in the Fig. 2); (2) For slag conditions with 10% and 3% FeO content, the prediction results of the theoretical decarbonization model deviate significantly. Therefore, the theoretical model needs to be improved.

## Improved decarburization comprehensive model of expansion droplet

For the multiphase slag conditions, the impact of the solid phase ratio on the physicochemical properties of slag need be considered. In this section, the solid phase ratio was analyzed at different FeO contents, and the improved decarburization comprehensive model of expansion droplet was developed based on the solid phase ratio and data fitting.

### Analysis of solid phase ratio for slag

Thermodynamic calculations have revealed that under the experimental conditions of Fruehan ($T = 1713{\text{ K}}$, ${{w_{{{\text{CaO}}}} } \mathord{\left/ {\vphantom {{w_{{{\text{CaO}}}} } {w_{{{\text{SiO}}_{{2}} }} }}} \right. \kern-0pt} {w_{{{\text{SiO}}_{{2}} }} }} = 1.2$, $w_{{{\text{MgO}}}} = 12 \, \%$), when $w_{{{\text{FeO}}}} \le \, 10 \, \%$, the slag exists in a multiphase form with coexistence of solid and liquid phases. The solid phase ratio in the multiphase slag ($f_{{{\text{solid}}}}$) has a significant impact on the physicochemical properties of the slag^23^, resulting in a significant deviations in the predictions of the decarburization model.

**Fig. 3:**
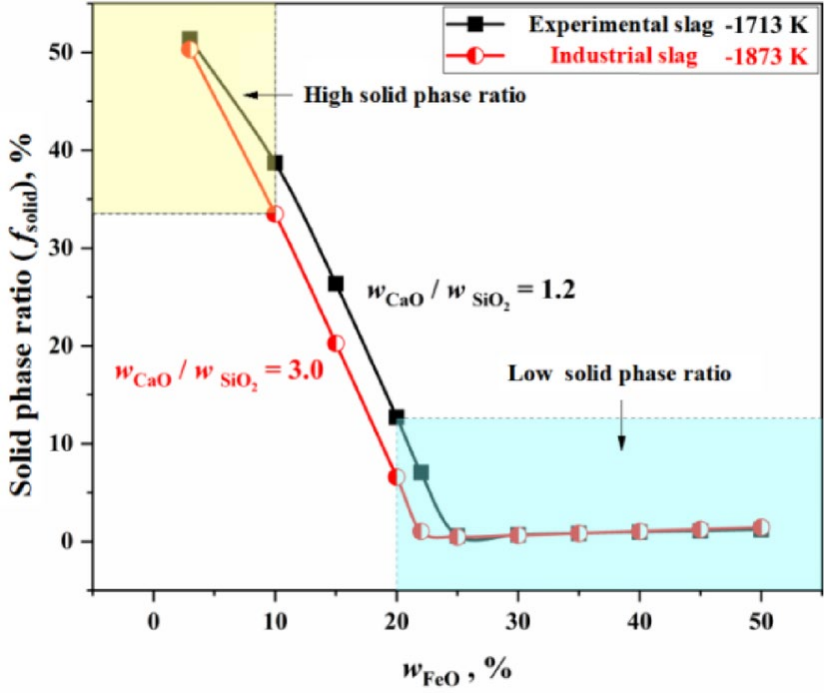
Solid phase ratio of experimental slag and industrial slag.

Figure 3 shows the solid phase ratio of the experimental slag of Fruehan and the industrial slag ($T = 1873{\text{ K}}$, ${{w_{{{\text{CaO}}}} } \mathord{\left/ {\vphantom {{w_{{{\text{CaO}}}} } {w_{{{\text{SiO}}_{{2}} }} }}} \right. \kern-0pt} {w_{{{\text{SiO}}_{{2}} }} }} = 3.0$, $w_{{{\text{MgO}}}} = 5 \, \%$) in this study, and the Equilib module of FactSage 7.0 thermodynamic software was used to calculate the solid phase ratio of the slag by minimizing the Gibbs free energy of the whole system.. In Fig. 3, it is evident that when the FeO content in the slag is greater than 20%, the solid phase ratio is low. Referring to the decarburization curves in Fig. 2, there is little difference in the reactivity performance of the two types slag under these conditions (the part above the droplet solidification line in the Fig. 2). However, when the FeO content in the slag is 10% or less, the solid phase ratio is higher, and correspondingly, there is a greater difference in the reactivity performance of the two types of slag, as shown in Fig. 2. This indicates that the solid phase ratio has a significant effect on the reactivity performance of slag.

### Establishment of improved decarburization comprehensive model of expansion droplet

For the multiphase slag in experiment or actual production, the effect of solid phase ratio on the physical and chemical properties of slag should be fully considered, so as to improve the droplet decarburization model. According to the study of Saito et al.^24^, the effect of solid phase ratio of slag on viscosity is shown in Eq. (8).

8 $$\frac{{\eta^{\prime}}}{{\eta_{0} }}{ = }\left( {1 - \frac{{f_{{{\text{solid}}}} }}{{f_{{{\text{solid}}}}^{*} }}} \right)^{ - 2.5}$$

where, $\eta^{\prime}$ is the viscosity of solid–liquid coexisting phase, Pa s; $\eta_{0}$ is the viscosity of liquid slag phase, Pa s; $f_{{{\text{solid}}}}$ is the solid phase ratio of slag, %; $f_{{{\text{solid}}}}^{*}$ is the critical solid phase ratio, when the viscosity is infinite. The value of the critical solid phase ratio in the literature is 74% based on the data obtained by viscosity measurement and SEM observation.

According to the generalized Stocks-Einstein equation^25^, the diffusion coefficient of a solution is inversely proportional to the viscosity. Therefore, the relationship between the revised diffusion coefficient in the multiphase slag ($D_{{{\text{FeO}}}}{\prime}$) and the diffusion coefficient of the pure liquid phase ($D_{{{\text{FeO}}}}$) is shown in Eq. (9).

9 $$\frac{{D_{{{\text{FeO}}}}{\prime} }}{{D_{{{\text{FeO}}}} }} = \frac{{\eta_{0} }}{{\eta{\prime} }}{ = }\left( {1 - \frac{{f_{{{\text{solid}}}} }}{{f_{{{\text{solid}}}}^{*} }}} \right)^{2.5}$$

Meanwhile, due to the presence of solid phase, the reaction interface area between droplet and slag in the model is also different from that of the whole liquid phase, which needs to be corrected according to Eq. (10)^26^.

10 $$\frac{{A_{{\text{d}}}{\prime} }}{{A_{{\text{d}}} }}{ = }\left( {1 - f_{{{\text{solid}}}} } \right)^{{{2 \mathord{\left/ {\vphantom {2 3}} \right. \kern-0pt} 3}}}$$

**Fig. 4:**
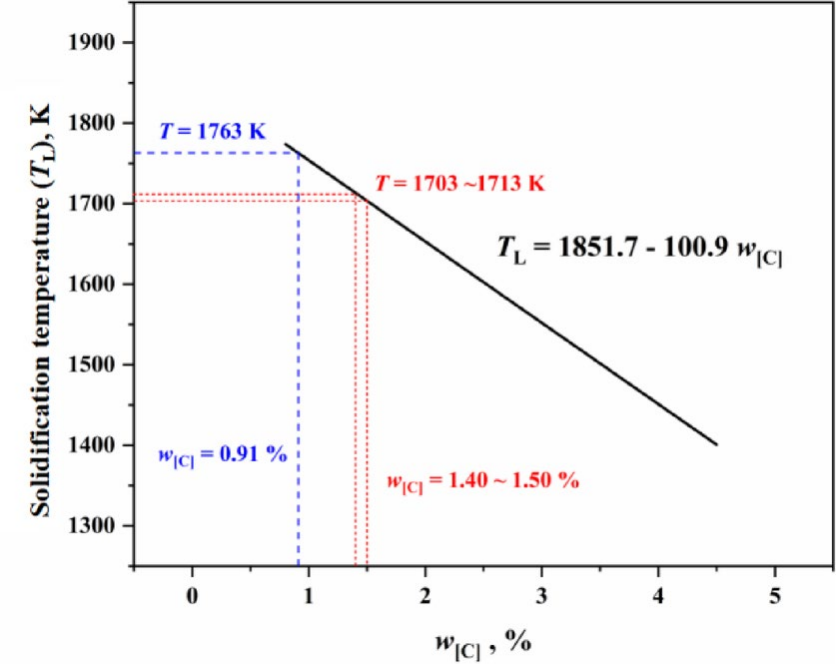
Relationship between the solidification temperature of molten iron and carbon content.

Equations (9) and (10) are substituted into Eqs. (2), (4) and (6) to obtain the improved droplet decarburization model based on solid phase ratio of slag, as shown in Eq. (11). Equation (11) is suitable for decarburization calculations when the droplets are in a liquid state. According to Fruehan’s research^16^, for slag with a high FeO content of 20%, the emulsion collapsed after approximately 8 s, causing the droplet to rapidly recoalesce and enter the thick slag layer, thereby marking the end of the fast reaction period. Simultaneously, Fig. 4 shows the relationship between the solidification temperature of molten iron and carbon content. According to the liquidus calculation formula for molten iron^27^, when the carbon content of the metal droplet decreases to 1.40–1.50%, the liquidus temperature rises to 1703–1713 K. At this liquidus temperature, the metal droplets solidify. The solidification of metal droplets not only hindered the continuous progress of decarburization reaction but also weakened the decarburization reaction, causing the droplet to end the suspension in advance and fall back into the bulk slag. It is evident that under high FeO slag conditions (with FeO contents of 20% and 30%), the droplet undergoes rapid decarburization and approaches the solidification line. Equation (11) is not applicable to the solidified state of the droplet. Based on the hot state experimental data of Molloseau and Fruehan^16^, Brooks et al.^19^ obtained the general relationship between droplet carbon content and time through nonlinear fitting, as shown in Eq. (12). Therefore, under slag conditions with FeO contents of 20% and 30%, for the solidification of metal droplets, Eq. (12) was introduced to develop a comprehensive model, as shown in Table 1.

11 $$\frac{{{\text{d}}w_{{\left[ {\text{C}} \right]}} }}{{{\text{d}}t}} = \left\{ { \, \begin{array}{*{20}l} {\frac{1}{50} \cdot \sqrt {\frac{{D_{{{\text{FeO}}}}^{{}} u_{{\text{d}}} }}{{\pi d^{3} }}} \cdot \left( {1 - \frac{{f_{{{\text{solid}}}} }}{{f_{{{\text{solid}}}}^{*} }}} \right)^{1.25} \cdot \left( {1 - f_{{{\text{solid}}}} } \right)^{{{2 \mathord{\left/ {\vphantom {2 3}} \right. \kern-0pt} 3}}} \cdot \frac{{\rho_{{\text{s}}} }}{{\rho_{{\text{d}}} }} \cdot \left[ {w_{{\left( {{\text{FeO}}} \right)}}^{{\text{b}}} - w_{{\left( {{\text{FeO}}} \right)}}^{*} } \right] \, ,} \hfill & {\left( {{{J_{{{\text{FeO}}}} } \mathord{\left/ {\vphantom {{J_{{{\text{FeO}}}} } {J_{{\text{C}}} }}} \right. \kern-0pt} {J_{{\text{C}}} }}} \right) \le 1} \hfill \\ {\frac{3}{25} \cdot \sqrt {\frac{{D_{{\text{C}}} u_{{\text{d}}} }}{{\pi d^{3} }}} \cdot \left( {1 - f_{{{\text{solid}}}} } \right)^{{{2 \mathord{\left/ {\vphantom {2 3}} \right. \kern-0pt} 3}}} \cdot \left( {w_{{\left[ {\text{C}} \right]}}^{{\text{b}}} - w_{{\left[ {\text{C}} \right]}}^{*} } \right),} \hfill & { \, \left( {{{J_{{{\text{FeO}}}} } \mathord{\left/ {\vphantom {{J_{{{\text{FeO}}}} } {J_{{\text{C}}} }}} \right. \kern-0pt} {J_{{\text{C}}} }}} \right) > 1} \hfill \\ \end{array} } \right.$$

12 $$w_{{\text{[C]}}} = a_{{\text{d}}} \cdot \left( {1 + e^{{ - k_{{\text{d}}} \cdot t}} } \right)$$

**Table 1:** Improved decarburization comprehensive model.

FeO content of slag	Mathematical formula describing droplet decarburization		
3% and 10%	Equation (11)		
20% and 30%	Equation (11)	Not solidification of metal droplets	Equation (11)
		Solidification of metal droplets	Equation (12)

where, *w*_[C]_ is the droplet carbon content; *t* is time; *a*_d_ and *k*_d_ are the fitting parameters. When the FeO content of the slag is 20%, the values of *a*_d_ and *k*_d_​ are 1.498 and 0.275, respectively; when the FeO content of the slag is 30%, the values of *a*_d_ and *k*_d_​ are 1.494 and 0.310, respectively. The values of *a*_d_ and *k*_d_​ were obtained through analysis and fitting using the Origin software.

### Evaluation of improved decarburization comprehensive model of expansion droplet

The performance of improved decarburization comprehensive model was evaluated using the Pearson correlation coefficient (PCC), mean absolute error (MAE), and root mean square error (RMSE), as shown in Eqs. (13)–(15)^28,29^.

13 $${\text{PCC}} = \frac{{\sum\limits_{i = 1}^{n} {(y_{i}^{\exp } - \overline{{y^{\exp } }} )(y_{i}^{cal} - \overline{{y^{cal} }} )} }}{{\sqrt {\sum\limits_{i = 1}^{n} {(y_{i}^{\exp } - \overline{{y^{\exp } }} )^{2} } \sum\limits_{i = 1}^{n} {(y_{i}^{cal} - \overline{{y^{cal} }} )^{2} } } }}$$

14 $$text{MAE} = \sum\limits_{i = 1}^{{N_{P} }} {\left| {y_{i}^{{\text{cal}}} - y_{i}^{\exp } } \right|} /N_{P}$$

15 $$\text{RMSE} = \sqrt {\sum\limits_{i = 1}^{{N_{P} }} {(y_{i}^{{\text{cal}}} - y_{i}^{\exp } )}^{2} /N_{P} }$$

where, *N*_*p*_ is the number of data, *y *^*exp*^ is the experimental value, *y *^*cal*^ is the calculated value, and $\overline{{y^{\exp } }}$ and $\overline{{y^{{{\text{cal}}}} }}$ are the average value of experimental value and calculated value, respectively.

**Fig. 5:**
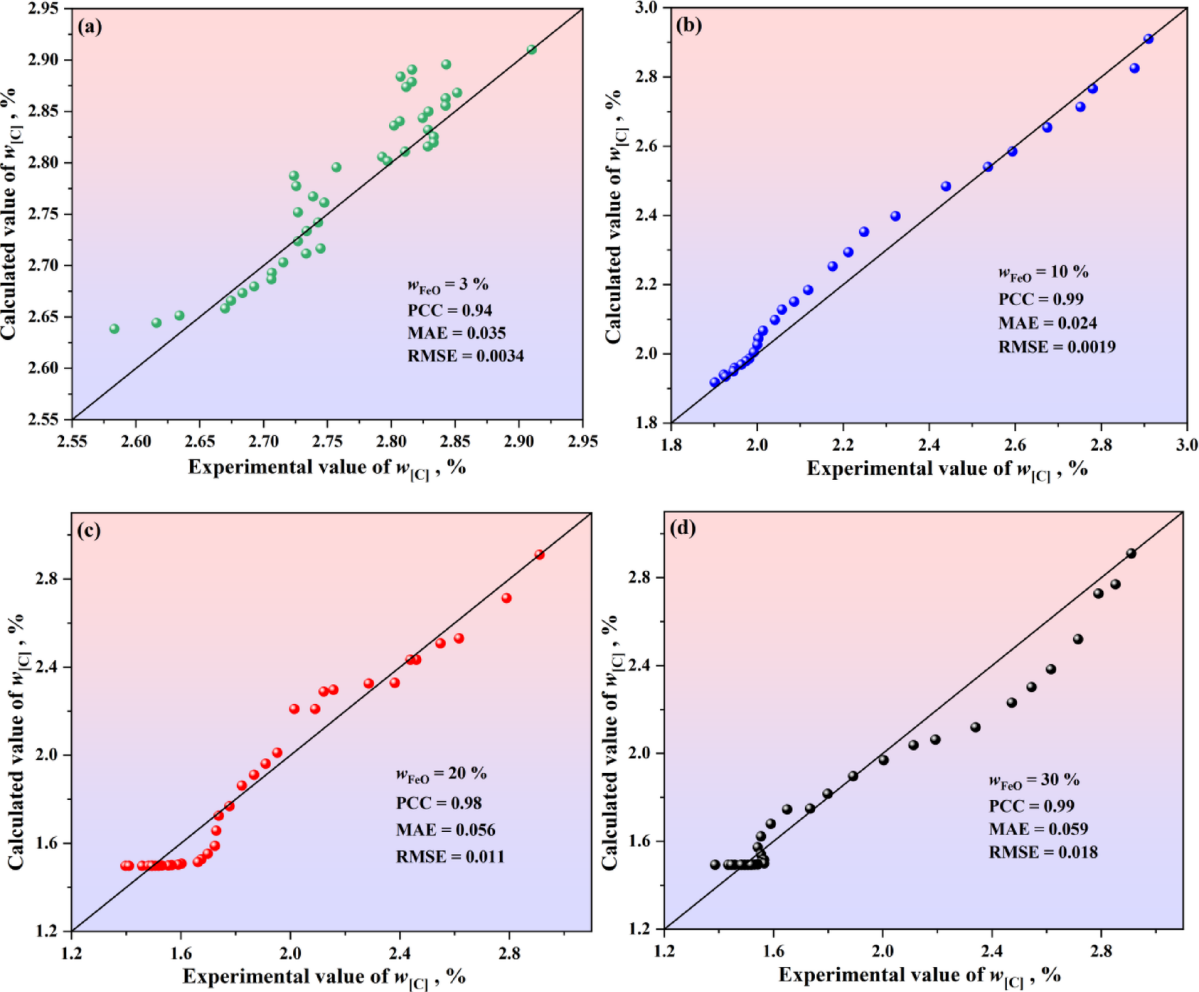
Comparison between experimental and calculated values obtained by improved decarburization comprehensive model of (**a**) *w*_FeO_ = 3%, (**b**) *w*_FeO_ = 10%, (**c**) *w*_FeO_ = 20%, and (**d**) *w*_FeO_ = 30%. The 45-degree diagonal dotted line is the identity where the experimental value is equal to the calculated value. The experimental value is sourced from Reference^16^.

To verify the calculation accuracy of the improved decarburization comprehensive model, the calculated values were compared with the experimental data for FeO contents of 3%, 10%, 20%, and 30%, as shown in Fig. 5. For FeO content of 3%, the model’s PPC, MAE and RMSE are 0.94, 0.035% and 0.0034%, respectively. For FeO content of 10%, the model’s PPC, MAE and RMSE are 0.99, 0.024% and 0.0019%, respectively. For FeO content of 20%, the model’s PPC, MAE and RMSE are 0.98, 0.056% and 0.011%, respectively. For FeO content of 30%, the model’s PPC, MAE and RMSE are 0.99, 0.059% and 0.018%, respectively. In addition, the closer the scatter to the 45-degree diagonal line, the smaller the error between the calculated and experimental values. Meanwhile, this figure shows that for FeO contents of 3%, 10%, 20%, and 30%, the scatter points are distributed around the 45-degree diagonal line, indicating the decarburization comprehensive model’s high prediction accuracy.

**Fig. 6:**
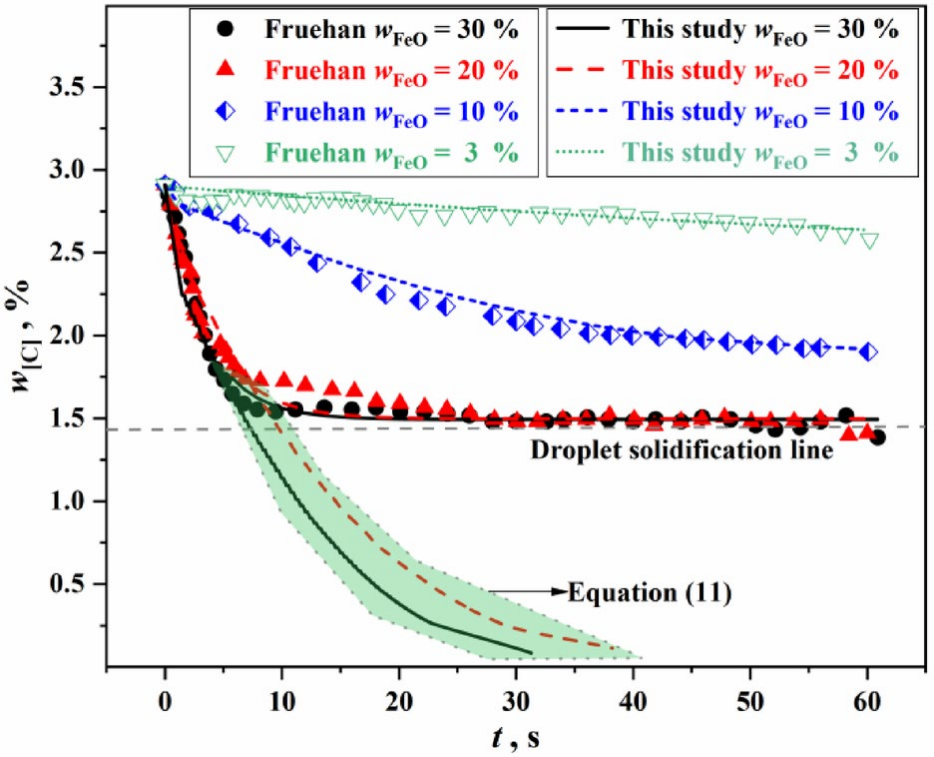
Calculation results of the improved decarburization comprehensive model.

Figure 6 shows the calculation results of the improved decarburization comprehensive model. It can be seen from Fig. 6 that for the slag with FeO content greater than or equal to 20%, the experimental data from Freuhan tended to plateau. This was mainly because the low experimental temperature caused the droplet to solidify after decarbonization, which affected the further progress of the decarburization reaction. The green shaded area in Fig. 6 represents the variation in carbon content of the metal droplets over time under the condition of no solidification of metal droplets. Compared to the theoretical decarburization model (as shown in Fig. 2), the improved decarburization comprehensive model provides calculations of carbon content changes in the decarburization reaction of Fe–C droplets in solid–liquid coexisting multiphase slag that closely match Freuhan’s experimental data. To sum up, the improved droplet decarburization comprehensive model proposed in this study can accurately calculate the change of carbon content of metal droplets under various complex multiphase slag conditions, overcoming the limitations of empirical data-fitting models that are applicable only to specific conditions and addressing the large deviations observed in the results of theoretical decarburization models.

## Conclusions

In this study, an improved droplet decarburization model based on solid phase ratio of slag and data fitting was established to calculate the change of carbon content. The main conclusions are as follows:

1. For slag conditions with 20% and 30% FeO content, the prediction results of theoretical decarbonization model are in good agreement with the results of Fruehan experiment (the part above the droplet solidification line); However, for slag conditions with 10% and 3% FeO content, the prediction results of the theoretical decarbonization model deviate significantly.
2. The solid phase ratio has a significant effect on the reactivity performance of slag. An improved droplet decarburization comprehensive model of slag was established, with all PCC exceeding 0.94 for different FeO contents, which is suitable for various multiphase slag conditions and better aligns with Fruehan’s experimental data.

## Data Availability

For data inquiries, please contact Zicheng Xin (sklxzc@163.con).
